# Post-Transfusion Occult Hepatitis B (OBI): A Global Challenge for Blood Recipients and Health Authorities

**DOI:** 10.5812/kowsar.1735143X.694

**Published:** 2011-09-01

**Authors:** Mohammad Kazemi Arababadi, Gholamhossein Hassanshahi, Ali Akbar Pourfathollah, Ebrahim Rezazadeh Zarandi, Derek Kennedy

**Affiliations:** 1 Department of Microbiology, Hematology and Immunology, Faculty of Medicine, Rafsanjan University of Medical Sciences, Rafsanjan, IR Iran; 2 Molecular Medicine Research Center, Rafsanjan University of Medical Sciences, Rafsanjan, IR Iran; 3 Department of Immunology, School of Medical Sciences, Tarbiat Modares University, Tehran, IR Iran; 4 School of Biomolecular and Physical Science, Eskitis Institute for Cell and Molecular Therapies, Griffith University Nathan, Queensland, Australia

**Keywords:** Hepatitis B, Transfusion, Epidemiology

## Abstract

Hepatitis B is one of the most frequent post-transfusion infections. Occult hepatitis B infection (OBI) is a form of hepatitis B infection in which, despite the presence of HBV-DNA in the serum and hepatocytes of the carrier, HBsAg is absent. In addition to the risk of transmission through the transfusion of infected blood, reactivation of hepatitis B in OBI patients and recipients of their blood can lead to cirrhosis, hepatic cancer, and reactivation of viral replication in the carrier. Therefore, effective assays to assess and screen for OBI in blood donors are of paramount importance and require urgent attention. Recently, several investigations in various regions of Iran have reported OBI in blood donors. In response, there has been a drive to apply more specific, sensitive, and accurate methods for the detection of HBV, which should become an obligatory screening process for all blood transfusion services. In this review, we address the progression of occult hepatitis B and the common problems associated with occult hepatitis B worldwide. Finally, we reflect on the research and screening that is being performed in Iran to deal with this problem.

## 1. Background

Hepatitis B Virus (HBV) is one of the most frequent and detrimental causes of liver infection in humans [1]. The most commonly noted symptoms of HBV-related hepatitis are jaundice, orangeade skin, and ophthalmia-protein, which is accompanied by increased bilirubin that results from the destruction of the liver cells that would normally process these proteins [2]. According to the latest reports, more than 2 billion people worldwide have serological markers of HBV, and more than 360 million of these are suffering from chronic HBV infection [3]. To decrease the risk of post-transfusion hepatitis (PTH) in the Iranian blood transfusion service (IBTS), all blood units and blood components are screened for HBsAg using ELISA [4]. Despite rigorous screening across the nation using modern equipment and internationally accepted screening methods to detect HBV, cases of PTH are still reported [5]. Hepatitis B infection is categorized into 5 clinical forms: [1] acute, [2] chronic, [3] fulminate, [4] asymptomatic, and [5] occult HBV infection (OBI) [6]. OBI is a form of long-term HBV infection; however, the clinical symptoms are undefined and differ from those of the previous described forms of HBV [7]. OBI differs from asymptomatic HBV infection in that although carriers are serologically negative for HBsAg, HBV-DNA is present in the serum [8]. Due to its particular properties, OBI is not detected by the common HBV assays, and it is thus capable of creating significant problems for blood transfusion services [9]. Therefore, in this review article, we consider different aspects of OBI, such as its prevalence in Iran and the throughout world, and assess the value of some serological diagnostic methods regularly used for OBI detection.

## 2. Clinical Symptoms Associated with OBI

Reactivation or low-level activity of hepatitis B virus in patients with OBI has been shown to lead to the development of other clinical disorders. Therefore, several research groups have focused on the correlation between hepatitis B infection and the onset of related clinical disorders. The clinical symptoms of OBI are mainly categorized into the following groups:

### 2.1. OBI and Chronic Liver Disease

It is well established that individuals who survive acute hepatitis B infection may shift to OBI [10], and despite regular examinations and clinical follow up for several years thereafter, mild necrotic inflammation can be observed in the liver tissue [10]. It could be speculated that, although OBI does not exhibit clinically distinct symptoms, the low-level viral replication and viral gene expression can initiate an immune response against hepatocytes, which is sufficient to generate liver necrosis [10]. Moreover, it has also been suggested that the severity of liver injury increases when chronic HCV carriers are co-infected with trace HBV particles by way of OBI [11][12][13], and that fibrosis and cirrhosis may occur in conjunction with OBI [14]. In general, it appears that any liver damage in the presence of residual HBV or OBI could initiate an immune response that can result in severe liver damage.

### 2.2. OBI and Hepatocellular Carcinoma

OBI is a possible risk factor for hepatocellular carcinoma (HCC) [15][16]. The following 3 situations have been well established as important in transducing OBI to induce HCC: 1) Merging of the HBV genome with a patient's chromosome, 2) Long-term inflammatory necrosis during OBI, and 3) cirrhosis that subsequently develops into OBI [16]. The exact steps required for this progression are not clear, and additional studies are required to draw a definitive conclusion regarding the effect of OBI in inducing HCC.

## 3. Transmission and Activation of OBI

OBI is considered a high-risk factor for both blood donors (due to the potential risk of hepatitis B reactivation) and recipients (due to the risk of infection transmission), which is described below.

### 3.1. Transmission of Infection

All OBI carriers are predisposed to transmitting HBV via blood transfusion and organ transplantation, especially liver allograft transplantation [17][18]. However, it should be mentioned, that as a result of the development of screening methods and programs for blood transfusion services worldwide, the incidence of PTH has decreased significantly. Despite these measures, some countries are still struggling with HBV transmission through these mechanisms [17]. For example, in the Asian region, several cases of PTH have been reported in India and Taiwan [19][20]. OBI transmission via organ transplantation has also been reported [18]. The risk of HBV transmission via OBI patients is enhanced in hepatic allograft engraftment since hepatocytes are the main source of HBV [21]. The risk of PTH from OBI patients is lower in heart [22] and renal transplantation [23], while bone marrow transplantation carries the lowest risk of PTH [24]. Some studies have reported that blood containing anti-HBc and anti-HBs, does not appear to transmit HBV components, even though the viral load ranges between 20 and 500 IU/mL [25]. For example, in a recent look-back study, 49 blood transfusion recipients were traced to 10 donors with OBI. However, the results showed that only 1 recipient had an HBV strain with 95% sequence homology to that of the donor [25], suggesting that the majority of transfusion patients that subsequently developed hepatitis B did not contract the disease via the OBI donor. Furthermore, they reported that after inoculation of 2 OBI donor serum samples into 4 chimeric mice, only 1 mouse subsequently had measurable HBV DNA [25]. This controversy needs further studies to be resolved.

### 3.2. OBI Reactivation

The unknown mechanisms that appear to suppress the replication and gene expression of HBV, along with the inability of the immune system to facilitate complete clearance of HBV are the primary reasons OBI becomes established [26]. It would appear that under any condition that leads to immune deficiency, such as AIDS or chemotherapy, HBV can be reactivated and commence a program of viral replication and gene expression that is subsequently followed by an active or fulminant form of hepatitis B infection [27][28]. The functional reactivation of HBV eventually leads to a systematic immune response of memory T cells against infected hepatocytes, which progresses to liver inflammation and viral hepatitis [28]. In addition to the abovementioned diseases and treatments that lead to suppression of the immune system, some background disorders, including hematological malignancies [29] and allograft transplantations of bone marrow and other organs [30][31], can cause OBI reactivation.

## 4. Prevalence of OBI among National and International Blood Donors

Published evidence indicates that OBI is spread widely throughout the world, and patients co-infected with chronic hepatitis C infection are at increased risk [10][32]. Moreover, investigators believe that several other factors, in addition to chronic HCV, may also contribute to the prevalence of OBI, including geographical, epidemiological, and ethnic factors [33]. For instance, we have not observed any cases of OBI among HCV-infected thalassemia and hemodialysis patients in the Kerman province of Iran [33][34]. In addition to HCV-infected patients, 45% of intravenous narcotic users [35] and 51% of patients with hemophilia [36] have been shown to suffer from OBI. A wide range of OBI (0-36%) has been reported in hemodialysis patients [11][13][34]. Altogether, these data indicate that the prevalence of OBI among blood donors varies from country to country in different parts of the world. For example, in the European countries of Poland, Italy, Spain, and Germany, OBI prevalence rates of 0.006%, 0.22%, 0.05%, and 0.0006%, respectively, have been reported [37] (Table 1). Our previous results in Isfahan showed that 9% of HBsAg- blood donors were HBV-DNA(+), while 1.43% of Rafsanjan blood donors were HBsAg-/HBV-DNA(+) [38]. Behbahani et al. and Amini et al. reported that 0.8% and 0.15% of HBsAg- blood donors in Shiraz and Tehran, respectively, were HBV-DNA(+) [39][40]. As described above, most studies evaluating OBI have been performed in blood donors, and only a small number of studies have evaluated OBI in the general population. For example, Song et al. reported that the rate of OBI in the general adult population of Korea was 0.7% [41]. Raimondo and colleagues researched OBI in individuals without hepatic disease who underwent liver resection or needle biopsy during abdominal surgery [10]. They showed that 1/6 of the Italian general population is a carrier of occult HBV infection [10]. In addition, Sharifi et al. reported that 3 out of 110 anti-HBc-positive family members of patients with acute or chronic HBV infection suffer from OBI [42]. Although there is minimal data available regarding the prevalence of OBI in the general population, based on the results of Song et al. [41] and Sharifi et al. [42], it seems that the prevalence of OBI in the general population is higher than that in blood donors. This may be related to the fact that blood donors are screened for HBV infection; therefore, it is detected as soon as possible and infected individuals are excluded from donation. In addition, it seems that the prevalence of OBI in blood donors has a regional variance, not only internationally but also nationally. In Iran, the infection rate ranges from 0.8% in Shiraz to 2.72% in Zahedan (Table 1). This national variance may be related to the fact that the prevalence of HBV infection is highest in Southeastern Iran [41], which has led to increased incidence of OBI, or perhaps the blood donors evaluated in our studies were more expansive than in other reports [4][43].

**Table 1:** Prevalence of OBI in Iran and Several Other Countries. The Table Shows the Occurrence of OBI in Blood Donors in Iranian Compared to that in Blood Donors in Other Counties. The Results Were Obtained from Several Research Groups, and the Appropriate References Are Cited.

	**OBI Condition, No.**	**Total, No.**	**OBI, %**
Iran			
Tehran [40]	3	2,000	0.15
Rafsanjan [43]	4	270	1.48
Shiraz [39]	16	2,000	0.8
Isfahan [43]	5	545	0.9
Rafsanjan [4]	57	3,700	1.54
Zahedan [42]	3	110	2.72
Poland			
Totals from different	17	250,191	0.006
geographical regions [37]			
Italy			
Rome [37]	8	35,016	0.02
Turin [37]	18	236,708	0.007
Messina [10]	16	98	16.3
Spain			
Barcelona [37]	8	15,545	0.05
Valencia [37]	12	117,829	0.01
Germany			
Frankfort [37]	7	1,348,759	0.0006
Mexican			
Mexico city [51]	17	158	0.006
Oman [52]	0	200	0.0
Brazil			
Brasilia [53]	5	150	3.3
South Korea			
Seoul [41]	7	1,047	0.7

## 5. Diagnosis of OBI

Obviously, the best method for the diagnosis of OBI is the examination of liver cells for the presence of HBV-DNA [10]. However, due to the invasive nature of biopsy and the possibility of undesirable injuries to the liver during preparation, most current OBI studies are performed on peripheral blood samples [10]. A patient who is HBsAg-/HBV-DNA(+) and entirely free of any clinical symptoms of hepatitis and jaundice is considered to have OBI [44]. Clinical researchers worldwide have paid special attention to the application of hepatitis B core antibodies (HBc-Ab; also referred to as anti-HBc) for the rejection of at-risk blood units and blood components in blood transfusion services [1]. HBc-Ab is well documented as the first detectable circulating antibody against HBV, which has the highest titer among antibodies [1]. Therefore, detection of this antibody is a useful confirmatory tool that can be used in parallel with HBsAg [44]. In some countries, including the USA and Japan, parallel screening for HBsAg and HBc-Ab is performed in all donor samples [17][45]; in contrast, in India, HBc-Ab is not in the screening program for blood transfusions [46]. A wide variety of studies have examined the detection of this antibody among Iranian volunteer blood donors. Our previous studies in Esfahan [47] and Rafsanjan blood donors [4][43] showed HBc-Ab frequencies of 8% and 5.18%-9.5%, respectively. Other research groups have reported the rates of HBc-Ab antibody in different cities of Iran, including 6.5%, 11.5%, 4.8%, and 4.8% in Shiraz, Tehran, Boroojerd, and Khoramabad, respectively [39][40][48]. The reasons for the discrepancies in these results from different teams of investigators in various parts of the world (Table 1) could likely be categorized as follows:

1. Different sample sizes in the studies

2. The use of different laboratory equipment and measurement kits with different specificities and sensitivities

3. Different racial, ethnic, and genetic backgrounds, which can often affect the humoral immune response to HBV, leading to differences in HBc-Ab production [49][50]

In conclusion, according to the abovementioned reports, it seems that the directors of Iranian blood transfusion services are in the best position to decide (based on scientific and economic criteria) how to implement regular screening for HBc-Ab in donated blood units with a view to reduce the risk of post-transfusion and post-surgery transmission of HBV.

## 6. Conclusion

Due to the high prevalence of HBc-Ab and OBI in Iranian blood donors, the authors of this article suggest a screening program for HBc-Ab with the use of commercial kits. We also propose the use of PCR-based screening programs for diagnosis of OBI in all IBTS.
